# Association between family functioning and academic burnout among vocational college nursing students: mediating role of academic self-efficacy

**DOI:** 10.3389/fmed.2025.1590280

**Published:** 2025-06-25

**Authors:** Yanying Yang, Sujiao Liu, Min Zhou, Lihong Miao, Xuejuan Liu

**Affiliations:** ^1^Department of Nursing, Henan Vocational College of Nursing, Anyang, Henan, China; ^2^School of Medicine, Quzhou College of Technology, Quzhou, Zhejiang, China; ^3^Medical Care School, Chengdu Polytechnic, Chengdu, Sichuan, China; ^4^School of Nursing, Changji Vocational and Technical College, Changji, China

**Keywords:** academic burnout, family functioning, academic self-efficacy, nursing students, mediating effect.

## Abstract

**Introduction:**

Academic burnout is not uncommon and interferes with the role shift from nursing students to qualified nurses, aggravating a shortage of workforce in global healthcare system. However, there is currently a lack of research examining the relationship between family functioning, academic self-efficacy, and academic burnout in nursing students. The purpose of this study is to examine the relationship among family functioning, academic self-efficacy, and academic burnout among nursing students.

**Materials and methods:**

This cross-sectional survey recruited 2,847 nursing students from a three-year vocational school located in Henan province in eastern China and used an online questionnaire to measure the participants’ basic information, family functioning, academic self-efficacy and academic burnout. Descriptive statistics, Pearson’s correlation, multiple linear regression and mediation analyses were used.

**Results:**

The mean (SD) score of academic burnout among nursing students was 53.98 (10.87). Family functioning (*r* = −0.39, *p* < 0.001) and academic self-efficacy (*r* = −0.64, *p* < 0.001) were negatively correlated with academic burnout. Family functioning (*r* = 0.46, *p* < 0.001) was positively correlated with academic self-efficacy. At the same time, academic self-efficacy plays a partial mediating role (indirect effect = −1.009; 95% CI, −1.114 to −0.910) in the influence of family functioning on academic burnout.

**Conclusion:**

Nursing students with higher level of family functioning may have a stronger sense of academic self-efficacy, which could lead to a lower level of academic burnout. This finding provides important guidance for educational administrators to formulate targeted strategies to prevent academic burnout of nursing students.

## 1 Introduction

As the global population ages rapidly, nurses are playing an increasingly important role in healthcare systems (1). Meanwhile, in the context of the global health crisis, the continuous emergence of new medical cases and mutated viruses like COVID-19 has further intensified the demand for health human resources (2). Therefore, the urgent need to strengthen healthcare human resources is more obvious than ever before, which requires nurses not only to have a solid educational background and professional skills, but also to have a strong professional motivation and responsibility to effectively cope with increasingly complex medical challenges (3). The high incidence of academic burnout among nursing students, a major source of the nursing workforce (4), seems to exacerbate the gap between the growing demand for high-quality nursing services and the potential pool of qualified nurses (3, 5). In addition, nursing students who have experienced academic burnout are more likely to face higher turnover rate and job burnout after employment (6), which exacerbates the shortage of nursing labor force to a certain extent. At present, the global burnout rate among nurses has reached 11.23% (7). This phenomenon has a negative impact on the supply of workforces in the nursing industry and the quality of nursing services. Academic burnout refers to a state of extreme fatigue due to prolonged exposure to chronic stressors in the work environment, manifested by decreased cognitive and emotional regulation, psychological alienation, and concomitant depressive mood and complaints of non-specific physical discomfort (8). Existing studies have shown that nursing students have a high prevalence of burnout syndrome (9), which affects the training quality of nursing students to a certain extent (10). Academic burnout affects nursing students’ academic performance and satisfaction with higher education (11), and is closely related to common negative mental health issues such as anxiety, depression and stress (12, 13). Therefore, early detection of academic burnout of nursing students is helpful to take targeted intervention in time and reduce the adverse effects of academic burnout.

Family is an important factor influencing academic burnout among college students (14). Family functioning refers to the quality of social and structural interactions among family members and is closely related to the quality of harmony, cohesion and communication among family members (15). Good family functioning has a positive impact on the mental health of medical students, as well as on their attitude and developmental tendency (15, 16). Supportive family functioning can foster a healthy state of mind and positive attitude, thus lowering academic burnout among medical students (17–19). In China, vocational college nursing students are the main source of nursing workforce in the future. Different from undergraduate students, they receive three-year junior college education. The training emphasizes career orientation and practical skills. The courses focus on clinical operations, and the theoretical content is concise, enabling them to quickly adapt to job requirements (20). However, the influence of family functioning on academic burnout of vocational college nursing students has not been explored, especially the potential mediating factors in the association between family functioning and academic burnout.

In addition to family factors, personal factors such as academic self-efficacy are also important factors influencing academic burnout among nursing students (1). Academic self-efficacy is characterized by individuals’ judgment and confidence in their ability to successfully complete academic tasks, which is divided into self-efficacy of learning ability and self-efficacy of learning behavior (21). The level of academic self-efficacy directly determines the possibility of hard learning and is an important factor affecting learning motivation (22). Individuals with high self-efficacy will make more efforts when they feel there is a gap between their personal performance and their goals, while those with low self-efficacy will reduce their efforts (22). In the learning process, academic self-efficacy is the source of learning motivation and the regulator of cognition, which is positively correlated with college students’ academic performance (23) and negatively correlated with academic burnout of college students and nursing students (1, 24–26).

The above studies explored the influence of family functioning and academic self-efficacy on academic burnout. However, the mediating role of academic self-efficacy in the relationship between family functioning and academic burnout has not been clarified, especially in vocational college nursing students. Therefore, we hypothesize that (1) Family functioning is negatively correlated with academic burnout, (2) Academic self-efficacy is negatively correlated with academic burnout, (3) Family functioning is positively correlated with academic self-efficacy, (4) Academic self-efficacy mediates the relationship between family functioning and academic burnout.

## 2 Materials and methods

### 2.1 Design

A descriptive, cross-sectional design was conducted to examine the relationship between nursing students’ family functioning, academic self-efficacy and academic burnout.

### 2.2 Participants and procedures

This study was approved by the Ethics Committee of Henan Vocational College of Nursing (No: 2024ZD095, 5th September 2024), a cross-sectional survey was conducted from December 2024 to January 2025 to explore the relationship between family functioning, academic self-efficacy, and academic burnout among nursing students in China. The survey was conducted in a 3-year vocational college in Henan Province, Central and eastern China, using convenient sampling. The instructions and questionnaires are published through “Questionnaire Star,” a professional online survey platform. Inclusion criteria: first-year, second-year, and third-year nursing students who are at the school and agree to participate this study. Participants under the age of 18 have obtained the consent of their parents or legal guardians. All participants were informed of the purpose of the survey and how to complete it before filling out the questionnaire, and once participants agreed and scanned a specific QR code, they were able to access the filling page and respond. To ensure the validity of the questionnaire, the questionnaire was tested before it was used. To prevent participants from filling in the questionnaire repeatedly and missing questions, the same participant was limited to fill in the questionnaire once by means of IP address and mobile phone number, and each question was set to “mandatory.”

Henan Vocational College of Nursing is the only public full-time higher vocational nursing college in Henan Province specializing in nursing education. The college has a total enrollment of 7,238 nursing students, with 5,251 currently on campus. This questionnaire survey covered 3,000 nursing students, among which 2,847 provided valid questionnaires, and the effective response rate was 94.9%.

The Power Analysis and Sample Size Software (PASS) 2021 (NCSS, LCC. Kaysville, Utah, United States) was used for the estimation of sample size in our study (Formula *N* = [Z1−α/2σ/δ]2). The average score and standard deviation (σ) of academic burnout among nursing students were 55.35 (10.65) (3). The tolerable error (δ) in this study was 55.35% × 1.5%, thus the minimum sample size requirement for nursing students in a two-sided 95% confidence interval (*Z* = 1.96) was 633. However, considering a potential dropout rate of 10%, in order to ensure an adequate sample size, at least 704 nursing students were needed. Therefore, the final sample size of this study was 2,847, which was sufficient according to the previous hypothesis calculation.

### 2.3 Instrument

Data were obtained using four questionnaires as follows: the general information questionnaire, the academic burnout scale (ABS), the academic self-efficacy scale (ASES) and the family APGAR index (APGAR).

#### 2.3.1 General information questionnaire

General information was divided into demographic characteristics and academic-related data of the participants. The former included seven items about participants’ age, gender, grade, ethnicity, residence, the only child and romantic relationship. The latter included eight items about class leaders, like nursing major, single parent, left behind and family factors. Family factors included average family income, parental education.

In our study, “Like nursing major” was defined as nursing students have a positive sense of identification with the nursing career, are willing to actively engage in learning and practice, and gain a sense of achievement or satisfaction from it. “Single parent” refers to a family structure where a child is raised by only one parent, either the father or the mother. “Left behind” refers to the situation where one of the parents has been living in another place for an extended period before the age of 16.

#### 2.3.2 Academic burnout scale (ABS)

This scale was developed by Chinese scholars Lian et al. (27). The scale consists of 20 items, covering three dimensions: dejection, improper behavior, reduced personal accomplishment. It adopts the Likert five-point scoring method (1 = “very much not in line,” 2 = “not very much not in line,” 3 = “uncertain,” 4 = “basically in line,” 5 = “very much in line”). The scale score is the sum of the scores of each item, with a total score range of 20 to 100. A higher total score indicates a more severe degree of academic burnout (28). In this study, the Cronbach’s α coefficient of the total scale was 0.88, and the Cronbach’s α coefficients of the three dimensions were 0.87, 0.72, and 0.82, respectively. According to the established criteria, the definition of the total score of academic burnout is as follows: ≤ 60 points indicate no academic burnout, 61–80 points indicate mild to moderate academic burnout, and ≥ 81 points indicate severe academic burnout (27).

#### 2.3.3 Academic self-efficacy scale (ASES)

The scale, compiled by Liang (21), comes from China and includes two dimensions of self-efficacy of learning ability and self-efficacy of learning behavior. It contains 22 questions and 11 questions in each of the two dimensions. Questions 14, 16, 17 and 20 adopted the reverse scoring method, and the higher the score, the higher the academic self-efficacy (1). In this study, Cronbach’s α of the total score were 0.92, and the two subscale scores were 0.95 and 0.74, respectively.

#### 2.3.4 Family APGAR index (APGAR)

The family APGAR index (APGAR) was developed by Smilkstein (29). It is used to assess the family functioning of the subjects (30, 31), and has been shown to have good reliability and validity in the research on the degree of family caring of Chinese students (15). It includes five parameters: adaptation, partnership, growth, affection and resolve. Three possible answers are allowed (“almost always,” “sometimes,” “hardly ever”), and the score ranges from 0 to 2 points. The point from each item is calculated to obtain the total score. Higher scores indicate better family functioning. Cronbach’s α of this scale in our study was 0.92.

### 2.4 Data analysis

First, basic data and academic burnout were described. Categorical variables were described as count and percentage. After normality test, numerical variables were described as mean ± standard deviation for those with normal distribution, and median (interquartile ranges) was used to describe those without normal distribution. We then compared the differences in academic burnout among different groups with different basic data. Pearson’s correlation analysis was used to explore the pairwise relationship between the total score and dimension score of academic burnout, family functioning and academic self-efficacy. Multiple linear regression was used to explore the relationship between each index and academic burnout. Finally, the mediation analysis was used to explore the mediating effect of academic self-efficacy on the relationship between family functioning and academic burnout. We used the total score and three dimensions of academic burnout as dependent variables to fit the model, and adjusted for the demographic factors with significance as confounding factors. The Bootstrap method was used to fit 95% CI and calculate the indirect effect. R (4.4.1) was used for all statistical analyses, and bilateral *p* < 0.05 was considered statistically significant. The R package “mediation” was used to complete the mediation effect analysis, and “ggplot2” was used to draw the correlation analysis diagram.

## 3 Results

### 3.1 Demographic data of the respondents

In this study, there were 2,847 participants, among whom nursing students ranged in age from 17 to 25 years, with a mean age of 19.0 years (SD = 1.1). A total of 2,312 (81.2%) were females. By grade, there are 1,672 freshmen (58.7%), 703 sophomores (24.7%), and 473 juniors (16.6%). Detailed demographic data are shown in Table 1.

**TABLE 1:** Academic burnout of nursing students with different sociodemographic characteristics (*N* = 2,847).

Variables	*N* (%) or mean (SD)	DE	IB	RPA	TAB
**Age**	19.0 (1.1)				
**Gender**					
Male	535 (18.8)	22.58 ± 6.85	16.89 ± 4.10	15.63 ± 4.62	55.10 ± 11.64
Female	2,312 (81.2)	21.35 ± 5.80	16.54 ± 3.68	15.83 ± 3.79	53.72 ± 10.67
*T*		3.852	1.821	−0.956	2.507
*P*		< 0.001	0.069	0.339	0.012
**Grade**					
Freshman	1,671 (58.7)	21.72 ± 5.59	16.99 ± 3.53	16.32 ± 3.64	55.03 ± 10.30
Sophomore	703 (24.7)	20.74 ± 6.27	16.03 ± 4.03	14.89 ± 3.99	51.66 ± 11.40
Junior	473 (16.6)	22.33 ± 6.97	16.11 ± 3.96	15.28 ± 4.61	53.71 ± 11.46
F		10.97	21.24	38.34	24.35
*P*		< 0.001	< 0.001	< 0.001	< 0.001
**Ethnicity**					
Han ethnicity	2,786 (97.9)	21.57 ± 6.03	16.61 ± 3.76	15.79 ± 3.95	53.96 ± 10.88
Minority ethnic groups	61 (2.1)	21.82 ± 6.31	16.61 ± 3.55	16.23 ± 4.35	54.66 ± 10.39
*T*		−0.301	−0.001	−0.791	−0.513
*P*		0.765	0.999	0.432	0.609
**Residence**					
Urban	569 (20.0)	21.59 ± 6.19	16.34 ± 3.87	15.45 ± 4.25	53.39 ± 11.37
Rural	2,278 (80.0)	21.57 ± 5.99	16.67 ± 3.73	15.88 ± 3.87	54.13 ± 10.74
*T*		0.079	−1.849	−2.189	−1.402
*P*		0.937	0.065	0.029	0.161
**The only child**					
Yes	193 (6.8)	21.23 ± 6.95	16.31 ± 4.14	15.34 ± 4.60	52.88 ± 11.58
No	2,654 (93.2)	21.61 ± 5.96	16.63 ± 3.73	15.83 ± 3.90	54.06 ± 10.81
*T*		−0.744	−1.033	−1.429	−1.378
*p*		0.458	0.303	0.154	0.169
**Romantic relationship**					
Yes	503 (17.7)	22.42 ± 6.17	16.78 ± 3.56	15.57 ± 4.06	54.78 ± 10.20
No	2,344 (82.3)	21.40 ± 5.99	16.57 ± 3.80	15.84 ± 3.93	53.81 ± 11.00
*t*		3.375	1.216	−1.336	1.907
*p*		< 0.001	0.225	0.182	0.057
**Class leaders**					
Yes	578 (20.3)	21.50 ± 6.32	16.09 ± 3.81	15.02 ± 3.92	52.61 ± 10.95
No	2,269 (79.7)	21.59 ± 5.96	16.74 ± 3.74	15.99 ± 3.94	54.32 ± 10.82
*T*		−0.342	−3.652	−5.308	−3.369
*P*		0.733	< 0.001	< 0.001	< 0.001
**Like nursing major**					
Yes	2,157 (75.8)	20.84 ± 5.88	16.15 ± 3.71	15.21 ± 3.76	52.20 ± 10.57
No	690 (24.2)	23.88 ± 5.94	18.03 ± 3.55	17.62 ± 4.00	59.54 ± 9.86
*T*		−11.735	−11.985	−13.997	−16.715
*P*		< 0.001	< 0.001	< 0.001	< 0.001
**Plan to work as a nurse after graduation**					
Yes	1,223 (43.0)	20.79 ± 6.26	16.00 ± 3.87	14.98 ± 3.94	51.78 ± 10.99
No	69 (2.4)	24.45 ± 8.16	17.88 ± 4.03	17.01 ± 5.82	59.35 ± 11.71
Uncertain	1,555 (54.6)	22.07 ± 5.64	17.02 ± 3.59	16.383.75 ±	55.47 ± 10.39
*T*		23.97	29.71	47.52	49.96
*P*		< 0.001	< 0.001	< 0.001	< 0.001
**Left behind**					
Yes	763 (26.8)	21.99 ± 6.07	17.03 ± 3.88	16.07 ± 3.82	55.09 ± 11.17
No	2,084 (73.2)	21.43 ± 6.01	16.45 ± 3.70	15.69 ± 3.99	53.57 ± 10.73
*T*		2.172	3.606	2.337	3.26
*P*		0.029	< 0.001	0.019	0.001
**Single parent**					
Yes	228 (8.0)	22.15 ± 6.33	17.14 ± 4.07	16.01 ± 3.98	55.29 ± 11.26
No	2,619 (92.0)	21.53 ± 6.00	16.56 ± 3.73	15.78 ± 3.95	53.86 ± 10.83
*T*		1.423	2.08	0.847	1.849
*P*		0.156	0.038	0.398	0.066
**Monthly household income (CNY per capita)**					
< 2,000	841 (29.5)	21.68 ± 6.29	16.73 ± 3.71	15.88 ± 4.13	54.29 ± 11.19
2,001–3,000	989 (34.7)	21.53 ± 5.59	16.64 ± 3.73	15.75 ± 3.64	53.91 ± 10.45
3,001–4,000	554 (19.5)	21.51 ± 6.07	16.56 ± 3.87	15.81 ± 3.79	53.88 ± 11.08
> 4,000	463 (16.3)	21.60 ± 6.42	16.37 ± 3.77	15.72 ± 4.44	53.69 ± 10.92
F		0.127	0.997	0.224	0.363
*P*		0.944	0.393	0.88	0.78
**Father education**					
Below junior high school	659 (23.1)	21.55 ± 6.15	16.85 ± 3.78	15.98 ± 4.06	54.38 ± 11.01
Junior high school	1,319 (46.3)	21.65 ± 6.05	16.62 ± 3.74	15.74 ± 3.82	54.01 ± 10.93
Senior high school/technical secondary school	648 (22.8)	21.65 ± 5.65	16.54 ± 3.73	15.93 ± 3.96	54.13 ± 10.44
Above senior high school	221 (7.8)	21.05 ± 6.65	15.93 ± 3.84	15.18 ± 4.38	52.16 ± 11.19
F		0.527	2.801	1.712	1.64
*P*		0.756	0.016	0.128	0.146
**Mother education**					
Below junior high school	872 (30.6)	21.61 ± 6.09	16.73 ± 3.76	15.97 ± 3.99	54.31 ± 10.73
Junior high school	1,215 (42.7)	21.68 ± 6.04	16.69 ± 3.73	15.67 ± 3.77	54.04 ± 11.01
Senior high school/technical secondary school	551 (19.4)	21.25 ± 5.54	16.38 ± 3.74	15.81 ± 3.97	54.45 ± 10.39
Above senior high school	209 (7.3)	21.72 ± 6.89	16.17 ± 3.95	15.75 ± 4.69	53.64 ± 11.80
F		0.559	2.484	0.685	0.587
*P*		0.731	0.029	0.635	0.71

DE, dejection; IB, improper behavior; RPA, reduced personal accomplishment; TAB, total academic burnout.

All categorical demographic variables with > 3 categories were properly dummy-coded (k-1 variables) prior to univariate analyses.

### 3.2 Academic burnout

Overall, 703 students (24.7%) showed academic burnout, of which 688 (24.2%) reported mild to moderate academic burnout and 15 (0.5%) reported severe academic burnout. The total score of academic burnout was 53.98 ± 10.87. Among the three dimensions of academic burnout, dejection scored the highest (21.58 ± 6.03), improper behavior scored the second (16.61 ± 3.76), and reduced personal accomplishment scored the lowest (15.79 ± 3.95). Detailed data are shown in Table 2. In terms of the score of academic burnout, there were some differences in the total score of academic burnout and the score of each subscales among nursing students in different grades. The score of academic burnout of nursing students who dislike nursing major is significantly higher than that of nursing students who like nursing major. The specific data are shown in Table 1.

**TABLE 2:** Academic burnout among nursing students (*N* = 2,847).

Variables	Score	Items	Range
DE	21.58 ± 6.03	8	8–40
IB	16.61 ± 3.76	6	*60–30*
RPA	15.79 ± 3.95	6	*60–30*
TAB	53.98 ± 10.87	20	*20–93*

DE, dejection; IB, improper behavior; RPA, reduced personal accomplishment; TAB, total academic burnout. The italic values show the ranges of scores.

### 3.3 Correlation between academic burnout, academic self-efficacy and family functioning (*N* = 2,847)

**FIGURE 1:**
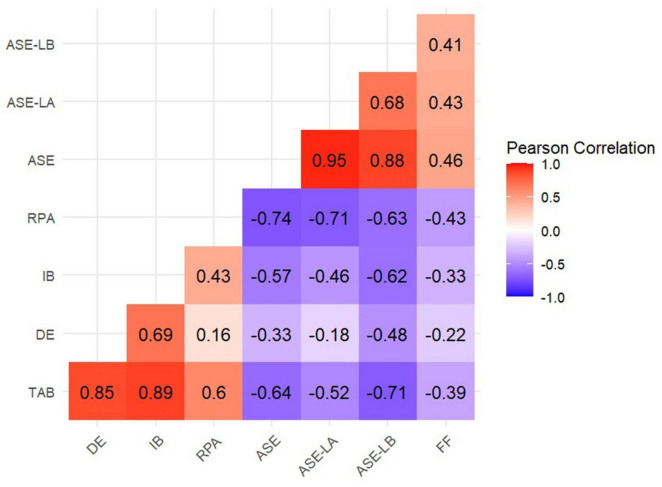
Correlation matrix of academic burnout, academic self-efficacy and family functioning. DE, dejection; IB, improper behavior; RPA, reduced personal accomplishment; TAB, total academic burnout; ASE, academic self-efficacy; ASE-LA, learning ability self-efficacy; ASE-LB, learning behavioral self-efficacy; FF, family functioning.

This study showed that academic burnout of nursing students was negatively correlated with academic self-efficacy (*r* = −0.640, *p* < 0.001) and family functioning (*r* = −0.389, *p* < 0.001). At the dimension level, the three dimensions of academic burnout were negatively correlated with the two dimensions of academic self-efficacy. Academic self-efficacy and its two dimensions were positively correlated with family functioning. The results of the correlation analysis are shown in Figure 1.

### 3.4 Multiple linear regression analysis of academic burnout

Multiple linear regression analysis showed that female, sophomore, like nursing major and interest in nursing occupation after graduation were significantly correlated with the total score of academic burnout. Family functioning (*B* = −0.39, *p* < 0.001) and academic self-efficacy (*B* = −0.52, *p* < 0.001) were independent influencing factors of academic burnout of nursing students. Table 3 shows that the model can explain 46.0% (adjusted R^2^:45.8%) of the variance in academic burnout among nursing students.

**TABLE 3:** Multiple linear regression analysis of academic burnout (*N* = 2,847).

Variable	B	SE	*t*	*p*	R^2^	F	*P*
					(Adj.R^2^)		
	96.511	1.153	83.681	< 0.001	46.00%	241.500	< 0.001
Gender (female)	−1.658	0.387	−4.278	< 0.001	−45.80%		
Grade (sophomore)	−1.505	0.365	−4.122	< 0.001			
Grade (junior)	0.559	0.425	1.315	0.189			
Class leaders (no)	−0.251	0.378	−0.663	0.508			
Like nursing major (no)	3.753	0.395	9.497	< 0.001			
Plan to work as a nurse after graduation (no)	2.533	1.043	2.428	0.015			
Plan to work as a nurse after graduation (uncertain)	0.335	0.329	1.017	0.309			
Left behind (no)	−0.622	0.341	−1.826	0.068			
ASE	−0.520	0.015	−35.184	< 0.001			
FF	−0.389	0.066	−5.891	< 0.001			

ASE, academic self-efficacy; FF, family functioning.

### 3.5 Mediation analysis

**FIGURE 2:**
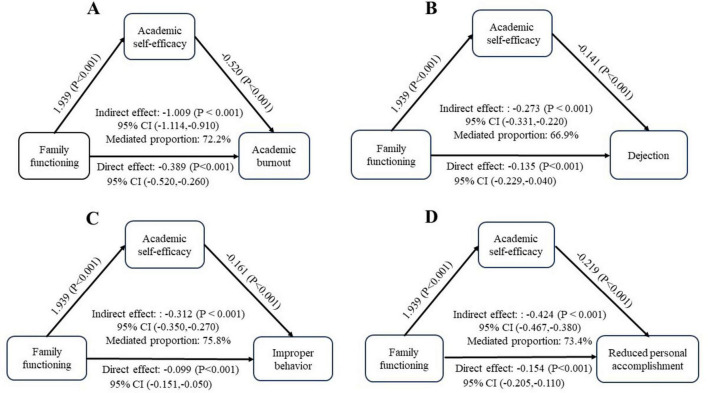
Path models showing academic self-efficacy mediating the relationship between family functioning and academic burnout. **(A)** Family functioning–> academic self-efficacy–> academic burnout; **(B)** family functioning–> academic self-efficacy–> dejection; **(C)** family functioning–> academic self-efficacy–> improper behavior; **(D)** family functioning–> academic self-efficacy–> reduced personal accomplishment.

After controlling for confounding factors such as gender, grade, whether to be a class cadre, whether to engage in nursing work after graduation, whether to like nursing and whether to have left-behind experience, academic self-efficacy partially mediated the relationship between family functioning and academic burnout score (indirect effect, −1.009; 95% CI: −1.120 to −0.900). In addition, academic self-efficacy partially mediated the relationship between family functioning and dejection (indirect effect, −0.273; 95% CI: −0.332 to −0.220). Academic self-efficacy partially mediated the relationship between family functioning and improper behavior (indirect effect, −0.312; 95% CI: −0.351 to −0.280). Academic self-efficacy partially mediated the relationship between family functioning and reduced personal accomplishment (indirect effect, −0.424; 95% CI: −0.469 to −0.380). The specific mediating effects are shown in Figure 2.

## 4 Discussion

This study is the first to explore the relationship among family functioning, academic self-efficacy and academic burnout among higher vocational nursing students in China. It is found that the higher the level of family functioning and academic self-efficacy, the lower the level of academic burnout of nursing students. At the same time, academic self-efficacy can mediate the effect of family functioning on academic burnout. In addition, gender, grade, class cadre, interest in nursing and left-behind experience all had a significant impact on the level of academic burnout of nursing students. Overall, the results of this study validated the proposed hypothesis.

In this study, the total score of academic burnout was 53.98 ± 10.87, which was similar to the research results of Wang et al. (3), but slightly lower than that of Zhou et al. (1). This study found that the academic burnout score of male students was higher than that of female students, which was consistent with the analysis results of Ye et al. (32). The possible reason is that nursing profession is regarded as a female-dominated field in the traditional concept, and male students may face pressure from peers and society to feel disapproved or maladapted in this field. The significantly higher scores for burnout among first-year students may stem from freshmen feeling overwhelmed by the multiple challenges they face as they transition from a stressful high school life to a college environment. The finding of higher levels of academic burnout among nursing students not serving as class leaders is consistent with previous findings (21, 33), and may be due to the fact that in China, The selection of class cadres is linked to an individual’s learning and management abilities (34), they gain a higher sense of self-efficacy through role identification, as well as more adequate social support and time management skills, which to a certain extent promotes academic performance and reduces the level of academic burnout (35). Nursing students who like nursing profession and are willing to be engaged in nursing profession have a low level of academic burnout. This may be because students who do not like or are uncertain about whether to engage in nursing profession in the future lack clear goals at the time of enrollment and feel confused about their future learning and development plans, which leads to negative learning attitudes and emotions, and long-term accumulation may further develop into academic burnout (36). This study also found that students with left-behind experience had higher scores of academic burnout. Students with left-behind experience are separated from their parents during the growth process and lack emotional support and care from their families. This lack of emotional support may cause them to feel lonely and helpless in the face of academic pressure, thus aggravating the generation of academic burnout (37).

This study found for the first time that family functioning of vocational nursing students was negatively correlated with academic burnout, which was consistent with previous research results on medical students and graduate students in China (15, 17). The complex and dynamic relationships that exist within families can be healthy and effective when family members live in harmony and maintain the integrity and functional autonomy of the family system. Conversely, when there is a lack of compromise among members, unhealthy and abnormal dynamics can occur (37). Family is an important source of support for students, and its functional emotional comfort helps to improve students’ physical and mental health, reduce anxiety and depression (15). A well-functioning family can be used as a protective factor to prevent academic burnout and help students develop appropriate autonomy and problem-solving strategies (38). Therefore, schools can compensate for the lack of family functioning of vocational nursing students by providing support to reduce their academic burnout. For example, schools could provide nursing students with relevant online course resources on family functioning so that students can learn strategies and techniques to improve family functioning. In addition, schools and classes can create a good campus culture by carrying out activities to promote school belonging and peer friendship, provide more school and peer support for nursing students, and enhance their sense of family belonging. Teachers can also provide more supportive interactions and care for nursing students during the teaching process to enhance their self-esteem and self-efficacy.

In addition, another finding of this study is that academic self-efficacy of vocational nursing students is negatively correlated with academic burnout, a finding consistent with previous research on college students, undergraduate nursing students, and vocational nursing students (1, 25, 26). In the learning process, academic self-efficacy is the source of learning motivation and the regulator of cognition (23). Students with high self-efficacy are more likely to set challenging, clear and specific learning goals, and persist in self-monitoring and self-management when they encounter obstacles. At the same time, people with a strong sense of self-efficacy can enhance positive emotions, such as motivation, sense of achievement and initiative, and these positive emotions help reduce the occurrence of academic burnout (1). Given that the academic self-efficacy of nursing students is not fixed, a series of methods (such as setting clear academic goals, observing positive role models, accepting social persuasion, and providing good teaching models) can effectively improve their self-efficacy (39). Therefore, nursing educators should consider developing relevant strategies to improve the academic self-efficacy of nursing students to reduce the risk of academic burnout.

The important finding of this study is that family functioning is positively correlated with academic self-efficacy in this group of vocational nursing students, which is consistent with the previous research results on junior high school students (40, 41). Parental participation in education affects the learning and interpersonal function of junior high school students, and then affects their positive perception of life satisfaction. They can improve their children’s educational achievements by encouraging and setting high expectations (42). When parents have a higher degree of academic participation, students’ academic self-efficacy also increases accordingly, and junior high school students with higher academic self-efficacy usually show better academic performance (40, 41). Another important finding of this study was that academic self-efficacy partially mediated the relationship between family functioning and academic burnout. Family functioning can not only directly affect the academic burnout of higher vocational nursing students, but also affect the academic burnout of nursing students through academic self-efficacy. This mediating effect was also established in the three dimensions of academic burnout, and improper behavior was the highest mediating percentage (75.8%). The findings of this study are consistent with those of previous studies. Middle school students or secondary vocational students with good family relations have a lower level of academic burnout (43, 44). When encountering adversity in learning, college students with high family cohesion and adaptability or healthy family views and values can adapt to the environment as soon as possible and find the most suitable learning method, greatly reducing the negative impact of academic burnout (45, 46). However, in the ineffective family emotional environment, parents’ negative response to children’s negative emotions will promote children to form uncontrollable emotional beliefs and low self-esteem, which will eventually affect the level of academic burnout of college students (14). It can be inferred that nursing students with good family functioning can reduce their academic burnout through positive academic self-efficacy. This finding helps us to better understand how family functioning affects academic burnout of vocational nursing students, and provides some effective intervention strategies.

### 4.1 Limitations

Our study firstly explored the mediating role of academic self-efficacy on the association between family functioning and academic burnout, thus providing new perspectives on interventions to improve academic burnout in nursing students. However, there are still several limitations. Firstly, the cross-sectional design of this study prevented the establishment of causality between the study variables. Therefore, future studies should consider longitudinal designs and more clearly reveal the dynamic relationships between variables. Second, the participants in this study were mainly from nursing students in a higher vocational college in Henan Province, central and eastern China, which did not cover representative nursing students in higher vocational colleges in various regions of China, which may affect the representability of the sample. Future intervention studies will be a worthy research direction, which will provide more empirical evidence for in-depth exploration of the related findings of family functioning, academic self-efficacy, and demographic variables on academic burnout.

## 5 Conclusion

Nursing students with higher levels of family functioning and academic self-efficacy showed lower levels of academic burnout, and family functioning may lower academic burnout through increasing the levels of academic self-efficacy. This provides important implications for educators to prevent and intervene with academic burnout among vocational college nursing students.

## Data Availability

The raw data supporting the conclusions of this article will be made available by the authors, without undue reservation.
